# Fabrication and Characterisation of Hydrogels with Reversible Wrinkled Surfaces for Limbal Study and Reconstruction

**DOI:** 10.3390/gels9110915

**Published:** 2023-11-18

**Authors:** Ryan L. Dimmock, Michael Rotherham, Alicia J. El Haj, Ying Yang

**Affiliations:** 1 School of Pharmacy and Bioengineering, Keele University, Stoke-on-Trent ST4 7QB, UK; 2 Healthcare Technologies Institute, Institute of Translational Medicine, School of Chemical Engineering, University of Birmingham, Birmingham B15 2TH, UK

**Keywords:** PDMS, wrinkled surface, reversible, limbal stem cell niches, biomimicry

## Abstract

In the biomedical field, there is a demand for the development of novel approaches for the investigation of optical epithelial anatomical features with biomimetic materials. These materials are not only required to replicate structures but also enable dynamic modelling for disease states such as limbal stem cell deficiency and ageing. In the present study, the effective generation of reversible wrinkled polydimethylsiloxane (PDMS) substrates was undertaken to mimic the undulating anatomy of the limbal epithelial stem cell niche. This undulating surface pattern was formed through a dual treatment with acid oxidation and plasma using an innovatively designed stretching frame. This system enabled the PDMS substrate to undergo deformation and relaxation, creating a reversible and tuneable wrinkle pattern with cell culture applications. The crypt-like pattern exhibited a width of 70–130 µm and a depth of 17–40 µm, resembling the topography of a limbal epithelial stem cell niche, which is characterised by an undulating anatomy. The cytocompatibility of the patterned substrate was markedly improved using a gelatin methacrylate polymer (GelMa) coating. It was also observed that these wrinkled PDMS surfaces were able to dictate cell growth patterns, showing alignment in motile cells and colony segregation in colony-forming cells when using human and porcine limbal cells, respectively.

## 1. Introduction

A new generation of materials is emerging with more of a focus on mimicking the biological, topographic, and bioactive features of the native tissue, such as the natural niche in which stem cells reside. Prominent examples include regenerative periodontal bioactive materials [1], skin-mimicking hydrogel dressings for advancing wound care [2], and enhancements for cellular integration in bone structural repairs [3]. In ophthalmology, biomaterials play a key role both surgically—in the form of stitches—and remedially (as contact lenses). When attempting to replicate the conditions of the ocular surface, it is paramount that not only the surface compatibility at the cell–surface interface is considered; the substrate’s stiffness and matrix replication are vitally important [4]. Biomaterials can be used to study the effects of changing the topography in relation to the stem cell niche. These materials can also be incorporated into bioreactor systems to define the role of the physical environment and stimulate mechanical, chemical, and electrical effects on the stem cell niche [5,6,7,8,9].

**Figure 1:**
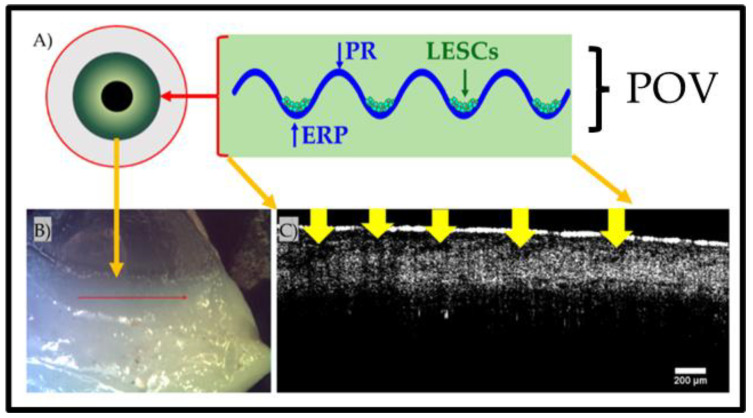
Location and structure of the limbal epithelial stem cell niche. (**A**) Illustration of the location and cross-section of the niche, (**B**) camera image of a human tissue segment with a red arrow denoting the OCT scan ROI, and (**C**) a B-scan OCT image with yellow arrows highlighting the ERPs within the POV crypt structure.

The cornea is the clear and transparent uppermost surface of the front of the eye. The corneal epithelium has multiple functions, with its primary function being to facilitate vision by allowing images to be transmitted to the retina at the back of the eye by permitting the transmission of light into the eye. Secondarily, the cornea, like all epithelia, exerts a barrier function to prevent infection of the globe. The cornea has a laminated structure that is comprised of the epithelial layer, the intermediate stroma, Bowman’s layer, and the endothelium (in order from the most environmentally exposed layer to the most internal layer) [10]. The limbal epithelial stem cell (LESC) niche presents as a crypt maintaining a renewable stem cell pool that can regenerate the growing cornea and facilitate repair through the expansion of LESCs from the limbus into the corneal epithelium [11]. The LESC niche is basally and suprabasally contained in the limbus, which is located circumferentially to the iris as the visible boundary of the sclera and the iris [12] (Figure 1). Within this circumferential crypt, undulations that are perpendicular to the ‘main’ crypt, are found; these are the Palisades of Vogt (POVs). As part of the POVs, the epithelial rete peg (ERP) forms the basal apex of the limbal palisades [13], whilst the palisade ridges (PRs) form the superior apex of each undulation [14]. In addition to these observations of their features, it has also been determined that LESCs reside in the basal apex of the crypts formed by the POVs [15]. These structures form the crypt-in-a-crypt structure that is unique to the limbus. These palisades segregate the LESCs in an anatomic stem cell niche that prevents the infiltration of aberrant cells into and out of the limbus and maintains a self-renewing stem cell pool [16,17]. In addition to the prevention of infiltration, the anatomical segregation of LESCs serves to ensure the LESC-to-corneal epithelial commitment in a controlled manner (preventing over-proliferative conditions, such as hyperplasia or cancer) by physically controlling LESC differentiation via mechano-transductive pathways. In particular, the YAP pathway is required to maintain the LESC phenotype [18]. 

Patterning of culturing substrates has been observed to have a significant impact on the localization, arrangement, differentiation, and cellular function of cells. The topography of substrates encompasses an enormous scope of different surfaces, ranging from variants of micropillar arrays to rough-etched nano-topographies and to wrinkled polymer substrates [19,20,21]. However, in the case of the limbus, few topographies can match the native architecture in vivo, which includes grooved and ridged topographies. An example is the creation of bioengineered limbal crypts that have been grown on moulded micro-ridges on a RAFT (Real Architecture for 3D Tissues) construct. This model demonstrated a high proportional yield of LESCs with the limbal stem cell marker p63α [22].

It has been established that static systems can maintain stemness [22,23], but what is not well defined is the use of dynamic systems in ocular applications. Dynamization holds great value for disease state modelling and has cross-applications for the prediction and correlation of glaucoma with limbal degradation [24]. However, to create a dynamic surface, the structure and the method for the creation of the topography must facilitate movement and manifest a biologically relevant change. As such, the surface wrinkling pattern, with various methods for generating soft polymer wrinkling as reviewed in [21], is a suitable candidate for the replication of the undulating anatomy of the limbal epithelial stem cell niche. 

In this study, polymer wrinkling was used to replicate the limbal niche architecture. Polydimethylsiloxane (PDMS) was selected as a bulk material due to its wide use in bioengineering applications [25,26,27]. PDMS is soft and highly deformable with a Young’s modulus that can be varied; therefore, it is ideal for use in producing biomimetic wrinkles [28]. Biocompatibility was also a key consideration. The inert surface of PDMS is not cytotoxic, making it ideal for cell culture, but it must be modified and/or coated with a protein matrix coating to achieve full cytocompatibility [29,30]. For the actual generation of the wrinkles, this bulk material must be modified to achieve a stiffness differential; for the surface to be wrinkled, it must be made at least an order of magnitude stiffer than the bulk material according to the simple wrinkling model as previously reviewed [21]. 

In this study, a novel method for generating a reversible wrinkle topography was defined. Additionally, the aim was to demonstrate a proof of concept in the biomimicry and modelling of the corneal limbal epithelial stem cell niche using a biomaterial platform that can dynamically undergo topographical conformational changes. In this endeavour, PDMS was modified to wrinkle using a dual treatment of acid oxidation and oxygen plasma exposure to create a biomimetic surface. Cytocompatibility was assured through the use of a photocured gelatin methacrylate (GelMa) coating. 

## 2. Results and Discussion

### 2.1. Stretching Frame for Creating a reversible Wrinkle Pattern on PDMS Substrates

**Figure 2:**
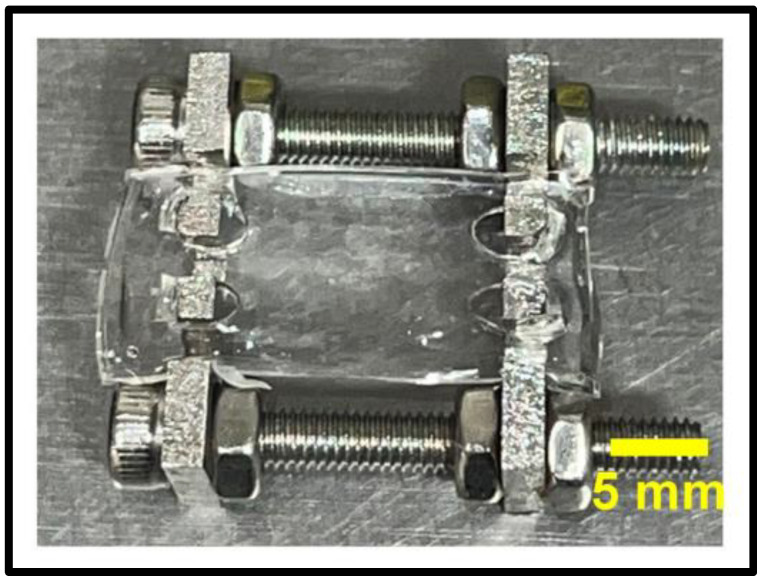
Photograph of the setup of the material treatment and stretching frame with an untreated PDMS chip suspended between the mounting pins.

First we developed a dynamic stretching platform consisting of a stretching frame that was specifically designed to both facilitate the treatment of the material treatment and alternately change the wrinkle pattern on the PDMS substrates. The stainless-steel frame, which is pictured in Figure 2, served multiple purposes—firstly, to pre-stretch the PDMS substrate chips (to 20% of the original length) before the respective material treatments. After the initial surface treatments, the wrinkle pattern remained undetectable whilst the strain was maintained on the suspended materials. Secondarily, the frame with the treated PDMS substrate was used to apply the GelMa coating while the substrate was stretched. The stretch application at this stage ensured that the GelMa coating covered all of the PDMS culturing surface, including all facets of the wrinkles that were formed after the frame was removed. Finally, the frame was used in culture with the substrates suspended between the pins in the “stretched” stages of the dynamic culture and served as a stabilizer for the PDMS substrate for initial cellular attachment. The frames were designed to be sterilized with an autoclave and to fit into a normal six-well culture plate. This size was chosen to facilitate ease of sterile access and manipulation of both the substrate and frame bolts with sterilised forceps and hex-keys. 

### 2.2. Imaging of the Wrinkled Substrates with Scanning Electron Microscopy

**Figure 3:**
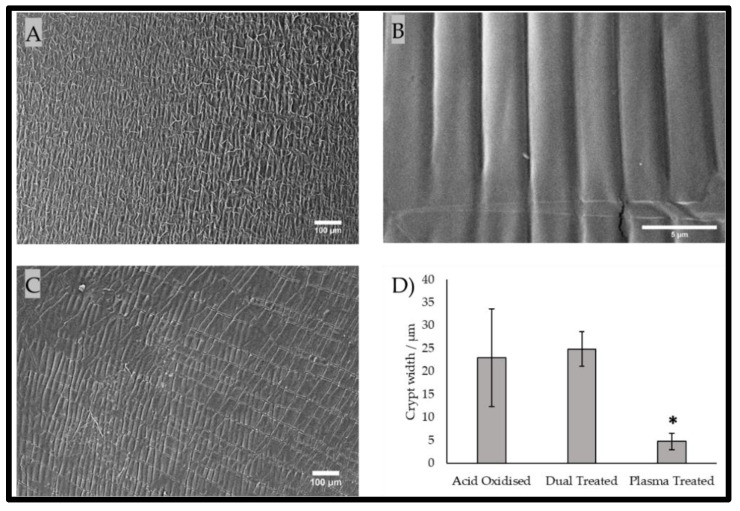
(**A**–**C**) Scanning electron micrographs of (**A**) acid-oxidised, (**B**) plasma-treated, and (**C**) dual-treated PDMS chips; scale bars are provided. (**D**) The wrinkle/crypt widths that evolved with each wrinkling method. Each bar represents a dataset (n = 3) according to its mean and standard deviation. * denotes statistical significance ascertained using the Games–Howell post hoc test of one-way ANOVA and α = 0.05.

Presented in Figure 3 are scanning electron micrographs for the three different PDMS treatment processes—from the singular plasma treatment and acid oxidation treatment to the combinatorial dual treatment. It was observed that there was a definitive difference in the wrinkle/crypt width among the treatment types. The narrowest features were found within the plasma-treated group, which exhibited a peak-to-peak crypt width of 4.73 µm ± 1.77 µm, whereas the largest was found in the dual treatment group, which had a crypt width of 24.85 ± 3.80 µm. The acid-oxidised group was fractionally narrower than the dual treatment group, but this group presented the greatest magnitude of error. The plasma-treated group was determined to be the only group that was statistically significantly different from the dual treatment group (but not the acid-oxidised group), with a *p*-value of 0.009, where α = 0.05. 

### 2.3. Mechanical Properties of the Wrinkled Substrates

**Figure 4:**
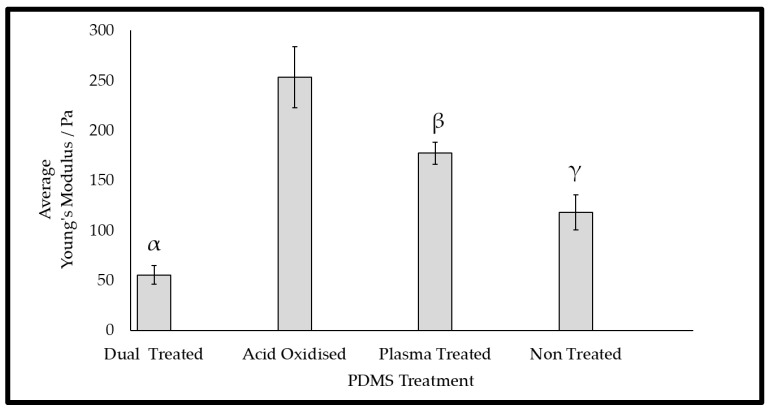
Results of tension tests on 3.3% PDMS chips that were exposed to the different wrinkling methods. Each bar represents a dataset (n = 3) according to its mean and standard deviation. The symbols α, β, and γ are used to signify groups that were found to be significantly different from each other, as ascertained using one-way ANOVA and employing the Games–Howel post hoc test; α, β, and γ were grouped where *p* < 0.05.

The mechanical properties (Young’s modulus) according to the tensile tests of the wrinkled PDMS substrates are presented in Figure 4. The results showed that the acid oxidisation and plasma treatment of the surfaces each led to a significant increase in the Young’s modulus, indicating stiffer substrates. In contrast, dual treatment of the surfaces significantly lowered the Young’s modulus in comparison with that of the non-treated surfaces, indicating that there was a softer surface after this treatment. It is proposed that the combination of the plasma treatment and acid oxidation increased the surface fragility, resulting in a lower average Young’s modulus. This was supported by the observation of anti-parallel linear surface fractures in the electron microscopy of the dual-treated surface (Figure 3C). This was further supported through the proposition of the mode of action and thickness of stiffening imparted by the different methods of treatment. The acid oxidation was supposed to permeate deeper during acid immersion, imparting an increase in bulk stiffness throughout more of the material [31]. During plasma exposure, only the upper surface was exposed, and it formed a very fine brittle layer, which was found to be prone to cracking [32]. In isolation, the plasma treatment conferred a palpable increase in stiffness, but in combination with a bulk that was pre-stiffened with the acid treatment, the crack faults became deeper splits, as observed in SEM (Figure 3C), and they penetrate deeply enough to truncate the results of the tension test, resulting in a lower Young’s modulus being reported. 

### 2.4. Depth Analysis of the Dual-Treated Substrates

**Figure 5:**
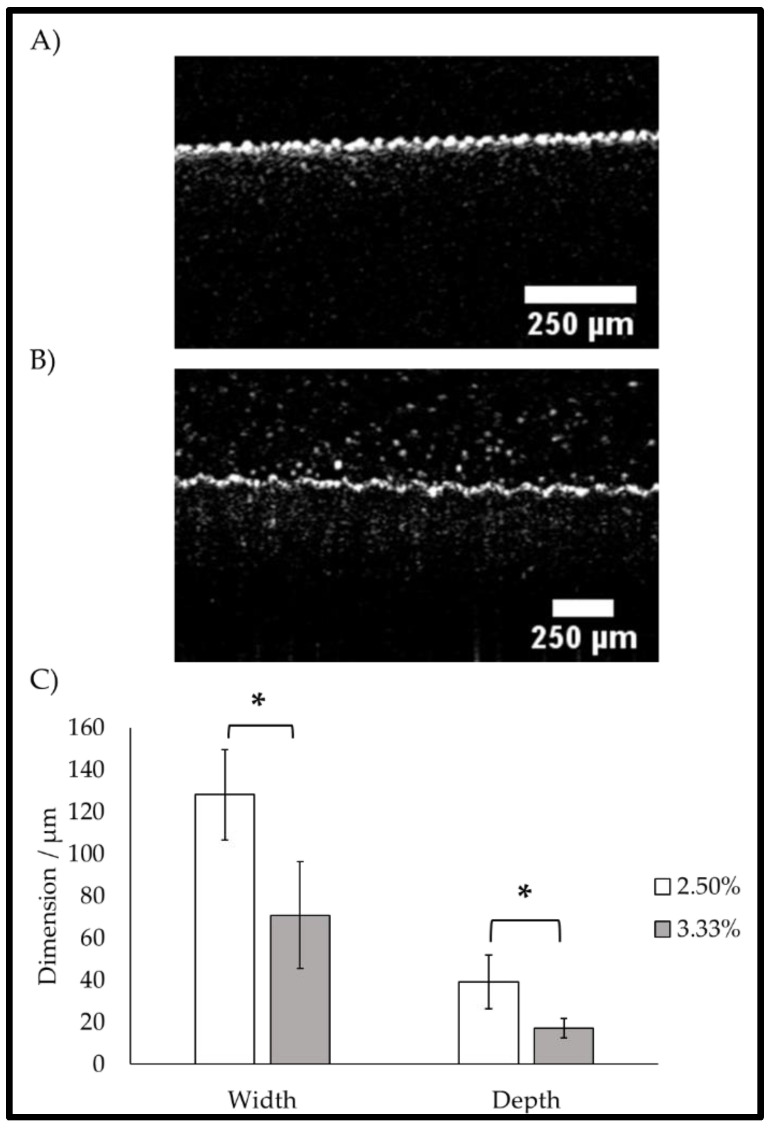
OCT images of the dual-treated PDMS chips. (**A**) PDMS substrate formulated with the 3.33% curing agent and (**B**) PDMS substrate formulated with the 2.5% curing agent; scale bars are presented in each image. Both images are representative of three replicated samples. (**C**) Bar chart describing the wrinkle/crypt dimensions acquired from B-scan OCT images using the mean (n = 3) and standard deviation of the dataset. * denotes statistical significance as ascertained with Student’s *t*-test where α = 0.05.

We next employed optical coherence tomography (OCT) to examine the cross-sectional features of the wrinkled surfaces. The OCT imaging of the dual-treated topography when hydrated revealed the ability of the dual treatment method to produce a repeatable crypt-like topography with distinct undulations (Figure 5). Additionally, it was demonstrated that the variation of the curing agent in the formulation of the PDMS substrate resulted in a change in the crypt dimensions after the dual treatment process. The OCT scans showed the crypt dimensions in terms of the peak-to-peak width and crypt depth through depth-resolved imaging. It was found that the respective peak-to-peak widths of the chips formulated with the 3.33% and 2.5% curing agents were 70.9 ± 25.39 µm and 128.2 ± 17.7 µm, respectively. The depths of the chips formulated with the 3.33% and 2.5% curing agents were 17.1 ± 4.64 µm and 39.1 ± 10.3 µm, respectively. Student’s *t*-tests were employed to assess the statistical significance of the differences between the curing agent concentrations while scrutinising both dimensions. It was ascertained that for both the width and depth, the curing agent concentrations significantly affected the crypt dimensions; for the peak–peak crypt width, the *p*-value was 0.033, and for the crypt depth, the *p*-value was 0.028, where α = 0.05. It was observed that the decrease in the curing agent concentration yielded an increase in both crypt width and depth. These crypt dimensions fell close to those measured in vivo using methods such as confocal microscopy, as established in the wider literature [13,33,34]. 

### 2.5. OCT of the Dynamization of Dual-Treated Substrates

**Figure 6:**
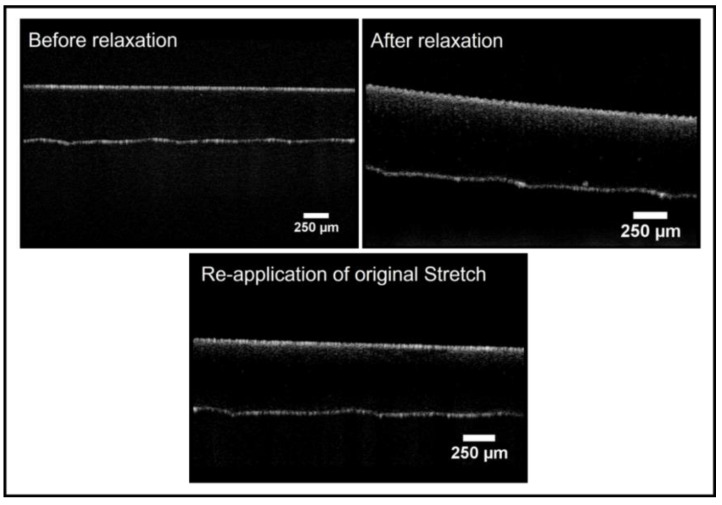
OCT images of a dual-treated PDMS (3.33% curing agent) substrate undergoing a cyclic load using the stretching frame. The B-scan cross-sections demonstrate the returnable nature of the dual-treated topography.

The wrinkle patterns on the PDMS substrates were then examined through OCT imaging while following cyclic stretch–release–stretch procedures (Figure 6). First, the PDMS substrates were subjected to the dual treatment protocol with the stretching frame in scan 1 (before relaxation); then, the substrates were removed from each stretching frame in scan 2 (after relaxation); finally, the stretching frame was reapplied to each of the substrates for the final scan (reapplication of original stretch). While this did not cyclically load the substrates for multiple cycles continuously, it clearly demonstrated the wrinkled topography to be reversible after formation. 

### 2.6. Human Primary Limbal Cells Seeded on Static Wrinkled Substrates

**Figure 7:**
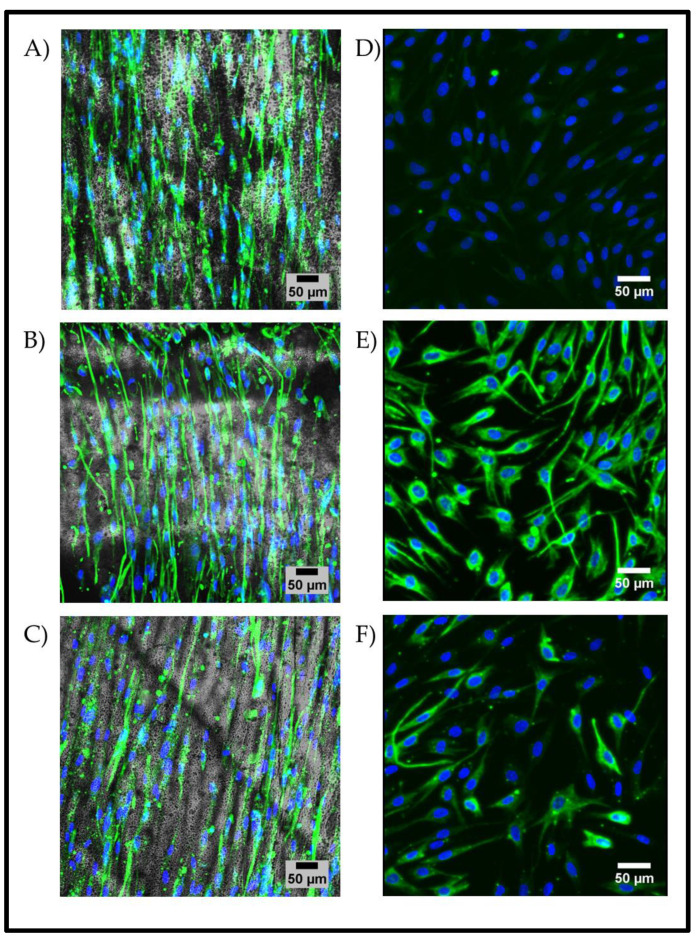
Immunofluorescent staining of primary human limbal cells cultured on static wrinkled PDMS substrates with the dual treatment and a 15% *w*/*v* GelMa coating (3.33% curing agent). (**A**) Positive staining for the epithelial marker cytokeratin-3 (green), (**B**) positive staining for the mesenchymal/transitional marker vimentin (green), and (**C**) positive staining for the neural/ectodermal marker nestin (green), which is associated with corneal cells. (**D**–**F**) Human limbal cells cultured on a tissue culture plate (TCP) as controls: (**D**) cytokeratin-3, (**E**) vimentin, and (**F**) nestin. The images are representative of n = 3; all scale bars represent 50 µm in length. Quantification of the directionality is shown in Supplementary Figure S1.

We then tested the ability of the wrinkled substrates to support and maintain primary limbal cells isolated from human limbal regions. Immunocytochemistry was used to assess the limbal cells’ morphology during culture on the wrinkled surface (Figure 7). The immunofluorescence of the limbal cells indicated changes in morphology, which could be attributed to the substrate topography’s influence on the cell shape through physical contact as a guidance mechanism. It was observed that the cells cultivated on the substrates adopted an elongated morphology that was aligned in parallel to the direction of the wrinkle ridges. In contrast, the monolayer controls on TCP had a multi-spindled morphology that was attributable to a mesenchymal cell type. The quantification of the alignment of the cells using ImageJ with the Directionality plugin and the local gradient orientation method confirmed this further (Supplementary Figure S1). A notable observation was the visible increase in the expression of the epithelial marker cytokeratin-3 on the wrinkled substrate compared to the control. The expression of vimentin (mesenchymal/transitional marker) and nestin (neural/ectodermal marker) did not fluctuate with the surface conditions. These motile, more fibroblast-like cells more readily conformed to the given topographies, demonstrating an ability to align in parallel with the direction of propagation of the wrinkles. The cells appeared to preferentially settle within the troughs of the wrinkles, altering their morphology from the random and multidirectional star shape to an aligned elongated shape. 

### 2.7. Porcine Limbal Epithelial Stem Cells on Static Substrates 

**Figure 8:**
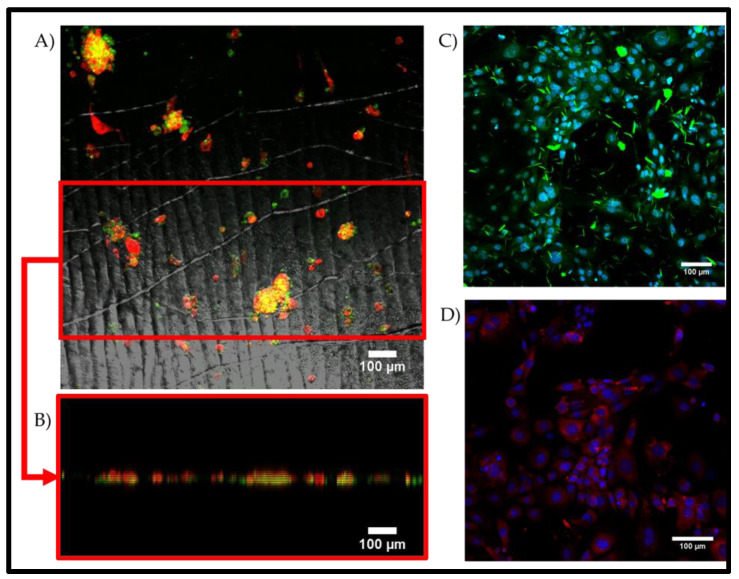
Immunofluorescent staining of primary porcine LESCs seeded on 2.5% static wrinkled PDMS with the dual treatment and a 15% *w*/*v* GelMa coating (2.5% curing agent). (**A**) The clustered colonies showed positive staining for the stem cell marker p63 (red) and the epithelial marker cytokeratin-3 (green). (**A**,**B**) The red ROI demarcates the region where the projection image was cut and rotated to obtain the XZ projected view, as demonstrated. (**C**) Control monolayer culture of porcine LESCs expressing CK3 (green) and (**D**) porcine LESC control culture expressing p63 (red). The images are representative of n = 3, and the scale bars are equal to 100 µm.

We also tested the ability of the substrates to maintain porcine limbal epithelial stem cells. The porcine limbal epithelial stem cells were isolated and transferred to the substrates at P0. During the cultivation on the substrates, it was observed that the colonies of cells adopted a more concentrated and bulbous shape when compared to the colony shape observed in the monolayer culture (Figure 8). Additionally, it was observed that the cells cultured on the wrinkled topography maintained their stem cell phenotypes, as demonstrated by their p63 expression patterns. 

### 2.8. Cellular Response to the Reversible Wrinkled Substrate 

**Figure 9:**
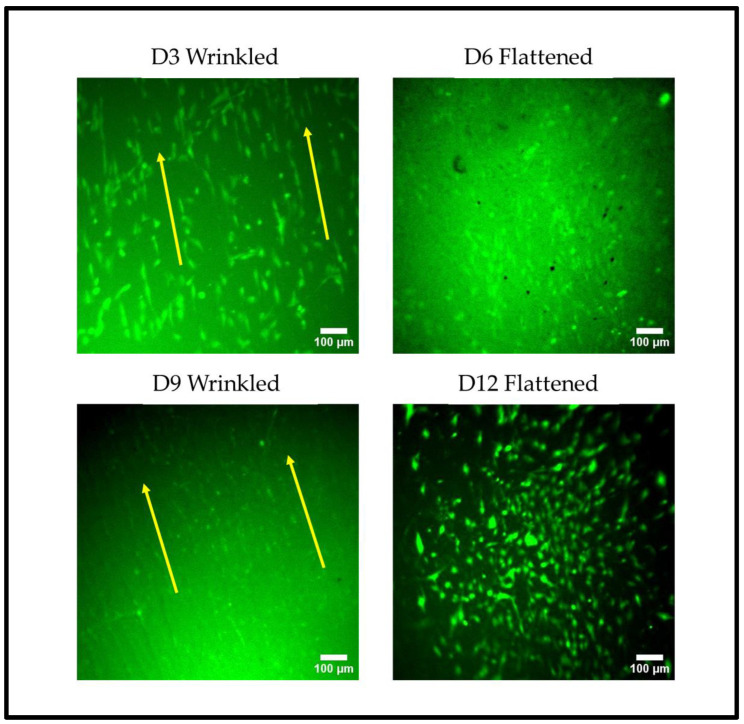
Cellular response to undulating wrinkled substrates in primary human limbal cells that were tracked live with Celltracker green. The green fluorescent images show the changes in cell morphology on the dual-treated and GelMa-coated substrates (3.33% curing agent), which were sequentially wrinkled, stretched to be flattened, relaxed, re-wrinkled, and re-flattened once more. The timeline is representative of three replicates, and the scale bars indicate 100 µm. The arrows indicate the linear winkle direction. Quantification of the directionality is shown in Supplementary Figure S2.

Human limbal cells showed a strong response to the wrinkle pattern. Hence, this facilitated the assessment of cellular alignment during dynamic topographic manipulation that was carried out mid-culture. To test the response of the cellular morphology to the reversable wrinkled substrate in the dynamic culture, the human limbal cells were tracked live by using the CellTracker green CMFDA live dye. During the relaxation stages, the substrates became wrinkled (D3 and 9), and the cells fell into alignment with the topography, which was evident through the uniformity in cellular elongation and parallel distribution. During the application of tension, the substrates became flattened (D6 and 12), and the cells adopted a more randomised distribution and less elongated morphology (Figure 9). The quantification of the alignment of the cells at D3 and D12 using ImageJ with the Directionality plugin and the local gradient orientation method also clearly confirmed this (Supplementary Figure S2).

## 3. Conclusions

In the present study, a novel reversible wrinkled cell seeding model that mimicked the native limbal epithelial stem cell niche was explored. A substrate with biomimetic features, including physiologically relevant crypt dimensions and suitable cytocompatibility, was created. It was demonstrated in preliminary limbal cell cultures that these wrinkled substrates were able to influence cellular morphology, growth patterns, and marker expression in static cultures. The model was also demonstrated to have capabilities for dynamic modelling, thus showing its great promise in the active manipulation of cellular morphology and growth patterns. The wrinkled PDMS surfaces explored in this study induced distinctly different cell growth patterns between cell types, showing alignment in motile cells and colony segregation in colony-forming cells. Thus, the ability to use this unique substrate system to characterise cell types according to their physical responses to contact-instructive topography was demonstrated. Some challenges still need to be addressed, including the isolation of human LESCs from stored tissue and the furthering of in-depth the characterisation of the cellular effects imparted by the topography and dynamization. However, this model presents a novel approach to the investigation of changes in topography with disease and ageing.

## 4. Materials and Methods

### 4.1. Preparation of PDMS Substrate

Polydimethylsiloxane (PDMS) was prepared from the two-component elastomer and curing agent Sylgard 184 kit (Dow Corning, Midland, TX, USA). The PDMS was prepared in 3.33% and 2.5% curing agent concentrations in elastomer, and it was thoroughly mixed and cast in 3D-printed chip moulds of fixed dimensions (10 mm wide, 20 mm long, and 0.5 mm deep). The mixture was cured in the moulds in a 65 °C oven overnight. After curing, the PDMS chips were released from the moulds and cooled down for further processing.

### 4.2. Preparation of the PDMS Substrate with a Reversible Winkle Pattern

**Figure 10:**
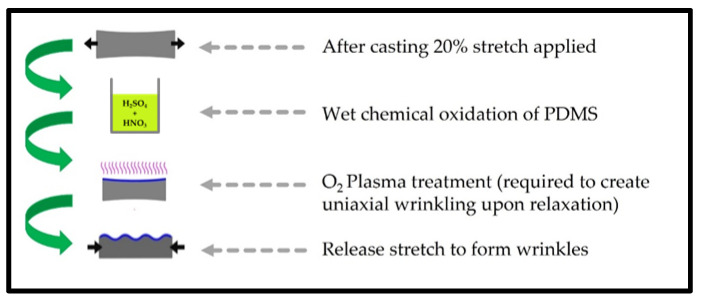
Schematic representation of the dual treatment protocol. These steps were undertaken to obtain the dual-treated PDMS substrates.

To create a reversible wrinkle pattern on the PDMS surface, the flat PDMS chips were suspended in stretching frames before acid exposure. The stretching frames were constructed from 316 L stainless steel, and the PDMS chips could be steadily fixed in the frames. The formation of wrinkles followed 2 procedural approaches, which were defined as single or dual treatment approaches (Figure 10). The single treatment referred to the performance of only oxygen plasma treatment or acid oxidation as follows. For acid oxidation, sulphonitric acid was prepared by mixing a ratio of 3:1 sulphuric:nitric acid, followed by heat treatment with stirring at 85 °C for 1 h. The PDMS chips were exposed to the prepared sulphonitric acid for 5 s, quenched in 1 M sodium hydroxide solution for 10 s, and then placed in a large water bath for 10 min to wash any un-neutralised chemicals from the surface/frames before being allowed to dry in air and released. Plasma-treated substrates were pre-stretched; the acid oxidation steps were omitted, and the substrates went through plasma exposure immediately before being released. 

For the dual treatment approach, the substrates were acid-oxidised as described; they were retained in low-temperature oxygen plasma (Diener, Ebhausen, Germany) for 10 min at 10 mbar O_2_ and 50 W of power. To finally allow the specimen to evolve into a wrinkled substrate, the treated material was released from the stretching frame. 

### 4.3. Gelatin Methacrylate (GelMa) Production and Coating 

GelMa was prepared to achieve a targeted 100% degree of substitution using 250 bloom gelatin dissolved in bicarbonate buffer at 55 °C with 0.938 mL of methacrylic anhydride per 100 mL of gelatin solution. The reaction mixture was stirred at 50 °C for 1 h before the reaction was terminated by adjusting the pH to 7.4. The reaction mixture was dialysed in distilled water for 3 days and then freeze-dried. The dried GelMa was re-constituted to 10% *w*/*v* in PBS and 0.25% *w*/*v* of lithium phenyl-2,4,6-trimethylbenzoylphosphinate (LAP) photo-initiator (Merck, Feltham, UK). Coatings were applied using a spreader on the substrates, stretched flat on the frames, and then photo-cured using a 405 nm curing lamp under aseptic conditions for 15 min. The substrates were then rehydrated using PBS and then sanitized using 70% ethanol for 30 min, followed by 3 washes with sterile PBS. During IMS sterilization of the GelMa-coated materials, the stretching frames were either replaced with pre-sterilized units or autoclaved before cell culture. 

### 4.4. Optical Coherence Tomography 

B-scans were conducted for the wrinkle-patterned materials using the Thorlabs spectral radar Telestro-II device (Thorlabs, Ely, UK). In the acquisition of the images, which was controlled through the dedicated Thor-labs OCT software, the pixel-averaging windows were set to 1 × 1 pixel, meaning that the highest-resolution images for each pixel were attained. Image field correction was applied to flatten the edges of the image to compensate for the lens distortion. The deepest clearly resolved depth was 600 µm. For volumetric images, the 3D mode was used to acquire multiple B-scans throughout a 6 × 6 mm acquisition area (XY). 

### 4.5. Scanning Electron Microscopy 

High-resolution imaging of the material surfaces was conducted by using a Hitachi TM4000 scanning electron microscope (Hitachi, Düsseldorf, Germany). The scanning parameters were as follows: an accelerating voltage of 15 kV, medium vacuum conditions, and use of the secondary electron (SE) detector. Before scanning, the samples were air-dried for at least 24 h before coating them with gold to increase their conductivity. The samples were sputter-coated with gold with a thickness of approximately 10 nm shortly before imaging. Statistical significance was determined by undertaking a Games–Howell post hoc analysis of a one-way ANOVA.

### 4.6. Tensile Testing

Tensile testing was performed on a Testometric mechanical testing machine (Testometric, Rochdale, UK); it was configured in the tensile arrangement with a 250 Kgf load cell fitted with PDMS chips suspended using custom suspension pins to fit the holes in the chips. The strain rate for the tensile tests was set to 0.1 mm/s. 

### 4.7. Cell Extraction and Culture 

Human cadaveric corneas were obtained from a tissue bank (National Health Service Blood and Transfusion Service, Bristol, UK), and fresh porcine eyeballs were obtained from a local abattoir (Staffordshire Meat Packers, West Midlands, UK). Limbal regions were microscopically identified in donor tissues before dissection; the most desirable regions presented prominent POVs, which were visualized as obvious radial striations. Limbal cells were extracted from dissected limbus tissue using a two-step enzymatic process. First, the limbus tissue portions were immersed in 1.2 IU/mL Dispase II for 2 h, and the epithelium was debrided. The debrided epithelium was then immersed in 1 mg/mL collagenase A overnight. Enzyme digests were obtained in an incubator maintained at 37 °C and 5% CO_2_. The digested epithelium was centrifuged once before being plated in DMEM/F12 medium containing 10% FBS, 1% penicillin/streptomycin, 20 mM L-glutamine, 0.1 X ITS supplement (Gibco, Scotland, UK), 1 µg/mL isoproterenol (Merck, Feltham, UK), 0.4 µg/mL hydrocortisone (Merck, Feltham, UK), and 2.43 µg/mL adenine (Merck, Feltham, UK). Limbal cells were seeded on the substrates in high-density droplets from passage 0 with isolation overnight (porcine) or immersion when seeded at 30,000/substrate (human). 

### 4.8. Immunofluorescence and Imaging 

Immunofluorescent stains were performed at the endpoint of culturing; the culture medium was fully aspirated and washed with PBS once, then subsequently fixed in 4% paraformaldehyde for up to 40 min. Fixed substrates were washed twice with PBS and then permeabilized with Triton-X 0.3% and blocked with 5% BSA. Human cells were stained with cytokeratin-3 mouse IgG (Abcam, Cambridge, UK) with the corresponding NL493 donkey anti-mouse IgG (R and D Biosystems, Abingdon, UK) alongside nestin and vimentin PE-conjugated mouse IgG (R and D Biosystems, UK) and 4′,6-diamidino-2-phenylindole (DAPI, Thermofisher, Dartford, UK). Porcine cells were stained with cytokeratin-3 mouse IgG and P63 rabbit IgG (Abcam, UK) with their corresponding donkey NL493 anti-mouse IgG and NL637 anti-rabbit IgG (R and D Biosystems, UK). The maximum projected images were captured using an Olympus FL2100 series confocal microscope (Olympus, Tokyo, Japan) and processed in ImageJ. To test the response of the cellular morphology to the revisable wrinkled substrate in the dynamic culture, human limbal cells were tracked live using the CellTracker green CMFDA live dye (ThermoFisher Scientific, UK) according to the manufacturer’s protocol. The cells were cultured on substrates that were sequentially wrinkled (Day 3), stretched to be flattened (Day 6), relaxed to the re-wrinkled state (Day 9), and re-flattened once more (Day 12). The cell morphology was imaged using a Leica MZ10F fluorescent dissecting microscope. Analysis and quantification of the alignment of the cells cultured on the substrates and in the controlled culture were performed post hoc using ImageJ with the Directionality plugin and the local gradient orientation method. 

## Data Availability

The data presented in this study are openly available in the article.

## Supplementary Materials

The following supporting information can be downloaded at: https://www.mdpi.com/article/10.3390/gels9110915/s1, Figure S1: Figure 1 (Imagej analysis to quantify the cell alignment on the wrinkled substrates, expressed as the number of features against the orientation in degrees in comparisonon the control cultures); Figure S2: Figure 1 (Imagej analysis to quantify the cell alignment on the wrinkled substrates (D3) and flattened substrate (D12), expressed as the number of features against theorientation in degrees).
