# Supplementary material for: Fabrication and Characterisation of Hydrogels with Reversible Wrinkled Surfaces for Limbal Study and Reconstruction
Source: Gels. 2023 Nov 18;9(11):915. doi: 10.3390/gels9110915 (PMC10671082; doi:10.3390/gels9110915)
Supplement: Supplementary file 1 [file gels-09-00915-s001.zip › gels-2701869-supplementary.pdf]

Supplementary Materials

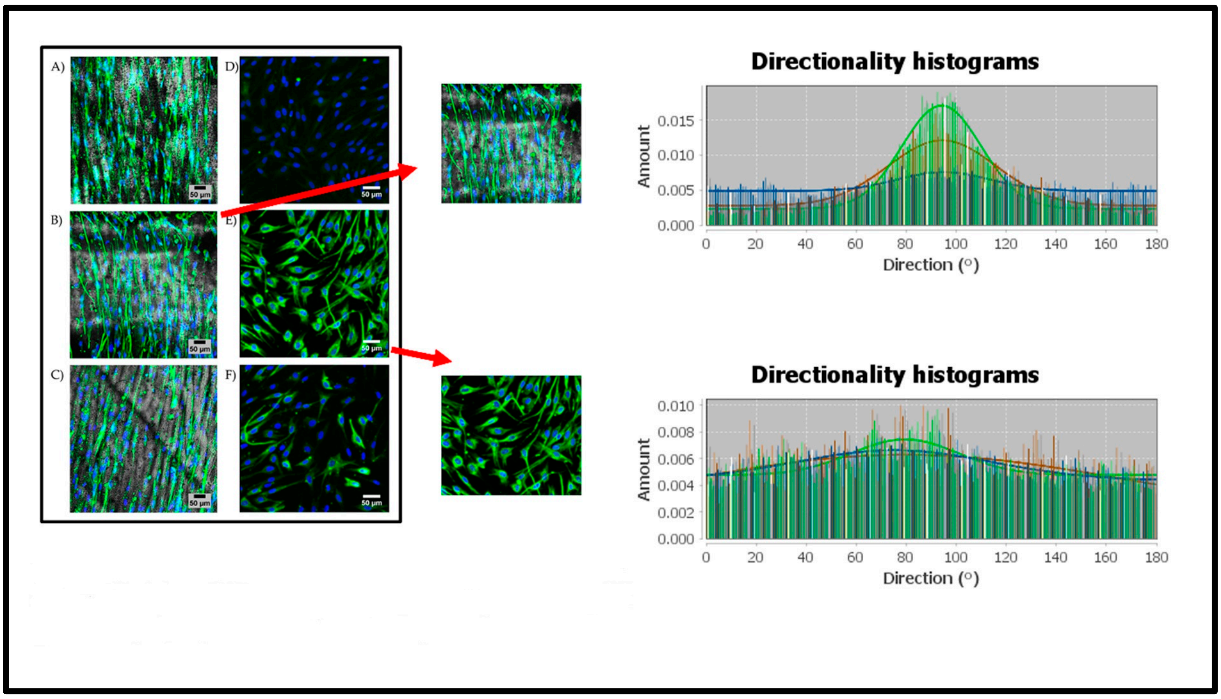

Figure S1: Imagej analysis to quantify the cell alignment on the wrinkled substrates , expressed as the number of features against the orientation in degrees in comparisonon the control cultures.

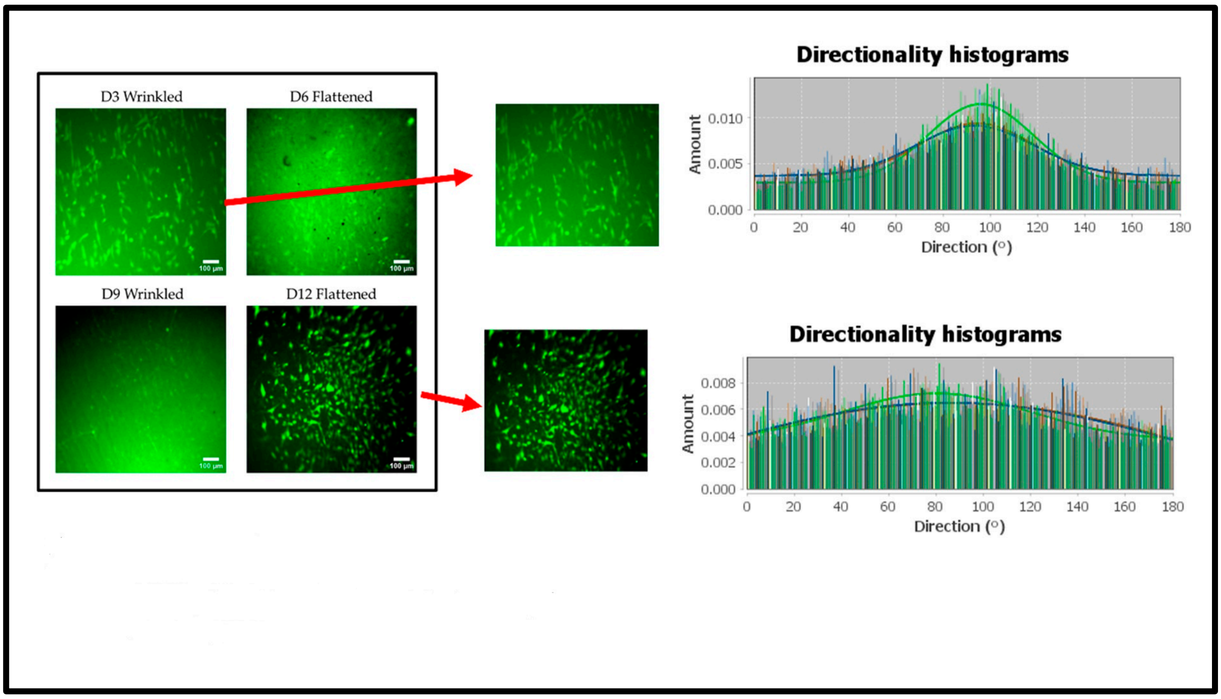

Figure S2: Imagej analysis to quantify the cell alignment on the wrinkled substrates (D3) and flattened substrate(D12) , expressed as the number of features against theorientation in degrees.
